# Comparison of ZETA Fast (PTS) (Optopol Technology) and Humphrey SITA Fast (SFA) (Carl Zeiss Meditec) Perimetric Strategies

**DOI:** 10.1155/2022/5675793

**Published:** 2022-02-03

**Authors:** Basil Mathews, Jeff Laux, Cassandra Barnhart, David Fleischman

**Affiliations:** ^1^Department of Ophthalmology, Kittner Eye Center at University of North Carolina, 2226 Nelson Highway, Chapel Hill, NC, USA; ^2^North Carolina Translational and Clinical Sciences Institute at University of North Carolina, 160 N. Medical Drive, Chapel Hill, NC, USA; ^3^Administrative Office, Bioinformatics Building at University of North Carolina, 130 Mason Farm Rd., Chapel Hill, NC 27514, USA

## Abstract

**Purpose:**

To compare two threshold strategies for visual field assessment, ZETA Fast (Optopol Technology) and Humphrey SITA Fast (Carl Zeiss Meditec), in controls and subjects with glaucoma. *Patients and Methods*. A prospective case-control study was carried out in which the clinical practice study included 26 controls and 26 glaucoma subjects. Testing for each strategy was monocular. Quantitative comparisons of mean deviation (MD), pattern standard deviation (PSD), visual field index (VFI), and test duration were made using two one-sided *t*-tests and Wilcoxon signed-rank tests. Confusion matrices were constructed to assess Optopol's detection as a proxy for Zeiss's detection of early glaucomatous defects. Receiver operating characteristic (ROC) curves were used to assess MD and PSD's discriminability.

**Results:**

The difference in MD values (Optopol-Zeiss) was within the margin for controls (difference = 0.36, *p*=0.06), but not for glaucomatous subjects (difference = 2.16, *p*=1.0). The Optopol strategy took longer than the Zeiss strategy in both controls (difference = 23 seconds, *p*=0.001) and glaucomatous subjects (difference = 49 seconds, *p* < 0.001). PSD values were higher and VFI values were lower from Optopol in glaucomatous subjects (*p* < 0.001 and *p*=0.002). Optopol was 92% sensitive in capturing early glaucomatous defects with MD <−2 when compared to Zeiss (*p* < 0.001). ROC analysis shows Optopol yields higher discriminability than Zeiss for MD/PSD indices.

**Conclusions:**

Both strategies enable effective identification of glaucomatous defects within 6 minutes; they also offer high sensitivity with a high correlation in global indices between the two strategies. The Optopol strategy is an alternative to the Zeiss counterpart with the limitation of a marginally longer testing protocol but a higher sensitivity of detecting glaucomatous defects.

## 1. Introduction

Automated perimetry remains the clinical standard for visual field assessment and determination of glaucomatous progression despite this era of rapidly evolving optical coherence tomography (OCT)-based diagnostic instrumentation [1–4]. It provides not only an estimation of retinal sensitivity and minimum threshold values to visual stimuli but also can confirm the functional effects of pathological findings discovered with other testing methods. Clinicians often use both visual field and OCT diagnostics together to identify a structure-function correlation or, in other words, to see if the pathological findings found on objective imaging correspond to visual field changes. Given that perimetry is an entirely subjective diagnostic test, it must provide accurate results within a relatively short time period but should also be readily accessible and affordable for use in broad screening.

As automation of perimetry gained popularity in the 1970s, the main goal of early investigators was to create an algorithm designed to capture the most sensitive threshold data [5–9]. The initial visual field approach that met these requirements was known as “bracketing” and presented a graded stimulus target of diminishing decibels to the subject until the minimum threshold was crossed and within a limit of error that was age-adjusted [10–12]. Although this was highly sensitive and accurate, it came at the expense of lengthy testing duration, which ultimately resulted in fatigue bias, lowering the reliability index, and utility of the test. Since advent, a variety of new perimetric testing strategies have been introduced with the intent of reducing testing time without sacrificing testing quality. One of the “new-generation strategies” which has gained great popularity and is now considered the clinical standard is the Swedish interactive thresholding algorithm (SITA) [13]. Initially, two SITA variants were developed in order to substantially shorten assessment duration without affecting data quality: SITA Standard and SITA Fast (SFA). After two decades of SITA strategy use with the Humphrey field analyzer (HFA, Carl Zeiss Meditec AG, Germany), another variant of SITA named SITA Faster (SFR) was introduced. This further reduced the test duration [14]. The manufacturers of modern perimeters are still working towards developing a strategy that yields reliable test results within the shortest possible time frame, with lowered costs of acquisition being a secondary but important consideration.

Optopol perimeters (Optopol Technology, Poland) operate using a proprietary testing strategy known as Zippy Estimation Thresholding Algorithm (ZETA) Standard and ZETA Fast, which also aim to reduce examination time without sacrificing result quality at reduced costs. The ZETA Standard uses Bayesian probability theory and probability density functions built from normative and statistical data. The company argues it is more robust in identifying accidental invalid responses by patients, leading to less nonspecific defects in healthy visual fields. This testing strategy leads to high accuracy as each location threshold is crossed at least once and the final threshold estimate is set at a probability function's peak, which is closer to the real subject's threshold. The ZETA Fast is a shorter version of the algorithm, which is analogous to HFA's SITA Fast. It requires at least one positive response to calculate the final threshold estimate which leads to its high sensitivity in detecting real defects as demonstrated below.

The aim of this study is to compare the results from the ZETA Fast perimetric strategy (Optopol Technology) against the SITA Fast algorithm (Carl Zeiss Meditec) in nonglaucomatous controls and subjects with glaucoma, showing that the ZETA Fast method is an equivalent and highly sensitive testing device.

## 2. Materials and Methods

This was a prospective study in which the participants comprised 26 healthy volunteers as controls and 26 subjects with glaucoma recruited from our clinical practice. Healthy subjects were defined as having no suspicious optic nerve head changes, no family history of glaucoma, intraocular pressures (IOPs) below 21 mmHg in at least 3 repeated tests with Goldmann applanation tonometry, no significant refractive error, and a best-corrected visual acuity better than 20/40; these patients had no prior visual field experience. Patients were required to have normal systemic blood pressures (systolic/diastolic readings below 140/90). Healthy volunteers were excluded if they had diabetes, cataract, corneal, or retinal disease. Further exclusion criteria included a family history of macular disease, glaucoma, or diabetes, ocular trauma, prior ocular surgery including laser surgery, unexplained visual loss, and diagnosed arterial hypotension and hypertension. The glaucoma group was defined as having glaucomatous optic nerve head changes (vertical quadrant thinning) with corresponding visual field defects that were attributed to intraocular pressures that were too high for the eye, no significant refractive error, best-corrected visual acuity better than 20/40, and prior experience of automated perimetry. If both eyes were eligible, a random choice was made to select the testing eye; this was to prevent including correlated results so that outcomes with lower reliability could be rejected. Each subject underwent 1 test each on the Optopol PTS perimeter and Zeiss HFA perimeter. Both devices used clinical standard testing parameters, comprising the following: Goldmann stimulus size III, white color, maximum intensity of 10000 asb, 300 mm aspherical bowl (consisting of 90° to the left and right, 60° superiorly, and 70° inferiorly), and background illumination of 31.5 asb. The tests on the Optopol PTS device were the ZETA Fast strategy, whereas the Zeiss HFA used the SITA Fast strategy. All tests were performed on the 24-2 test field, without the fovea and short-fluctuation (SF) testing. The order of tests was randomized to avoid fatigue bias. Only the tests which passed all reliability criteria were included. The criteria were as follows: false positive errors <25%, false negative errors <25%, and HK (blind spot) <25%. Additional criterion was to have central visual fields up to 22° without significant losses (not more than 2 points with sensitivity < −6 dB from the HoV).

The results were imported into R statistical software. The following information was extracted: test duration, mean deviation (MD) index, pattern standard deviation (PSD) index, and visual field index (VFI).

The primary hypothesis for this study was that the visual field mean deviation (MD) measurement from the Optopol machine did not differ from the Zeiss machine by more than 1 dB in either control subjects or subjects with glaucoma. This was assessed by using the two one-sided *t*-tests (TOST) procedure within each group [15]. Thereafter, we tested whether the time it took to conduct the tests (measured in seconds) and the differences in visual field pattern standard deviation (PSD) and visual field index (VFI) differed between the perimetric strategies within each group. Paired *t*-tests were preferred, but if the data were not sufficiently normal, normalizing transformations were used, if possible, or the Wilcoxon signed-rank test, if not. Bland–Altman plots were constructed for each combination of group and variable to assess agreement. Next, the ability of Optopol PTS perimeter with ZETA Fast to detect subjects whose MD was less than −2 was of particular interest. Considering the Zeiss machine as a clinical standard, confusion matrices associated with using the Optopol detection as a proxy for the Zeiss detection were created to determine how accurate the Optopol perimetric strategy was in identifying subjects with early defects with a MD of less than −2. To further characterize the confusion matrices, binomial tests were conducted to determine if the accuracy of the Optopol detection was greater than guessing the most prevalent outcome (i.e., no information rate), in conjunction with McNemar's test to assess bias. Sensitivity and specificity were provided for these findings. Lastly, receiver operating characteristic (ROC) curves were created to assess the ability of the two machines to distinguish between control and glaucomatous subjects based on MD and PSD. An alpha value of 0.05 was used for significance throughout this study. This study was conducted in compliance with both rules and regulations of the Health Insurance Portability and Accountability Act and the Declaration of Helsinki. Approval from the institutional review board (IRB) was obtained.

## 3. Results

A descriptive overview of our sample is provided in Table 1.

All glaucoma patients recruited for this investigation had prior history using the Humphrey visual field perimeter. None had used Optopol beforehand. None of the control patients had experience with either perimeter.

The difference between the machines' MD values (Optopol-Zeiss) was significantly within the margin for control subjects (difference = 0.36, 95% CI = (0.04, 0.76), d*f* = 25, *p*=0.06), but not for subjects with glaucoma (difference = 2.16, 95% CI = (1.52, 2.79), d*f* = 25, *p*=1.0). The latter data were nonnormal; thus, as a sensitivity analysis, logs were taken to normalize the data, and the transformed data were retested; the nonequivalence remained (difference = 1.11, 95% CI = (0.92, 1.29), d*f* = 25, *p*=1.0). Using the Optopol machine, the test took significantly longer than using the Zeiss machine in both control subjects (mean difference of 23 seconds, *p*=0.001) and subjects with glaucoma (mean difference of 49 seconds, *p* < 0.001).

**Figure 1:**
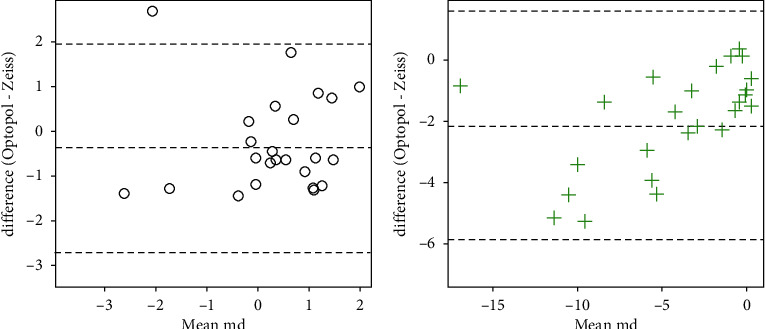
Bland–Altman plots for the visual field mean deviations. For each subject, the mean of the measurement from the Zeiss and Optopol machines is taken and plotted on the *x*-axis. The *y*-axis is the difference between the Optopol and Zeiss measurements. The plot on (a) is for control subjects; the plot on (b) is for subjects with glaucoma.

**Figure 2:**
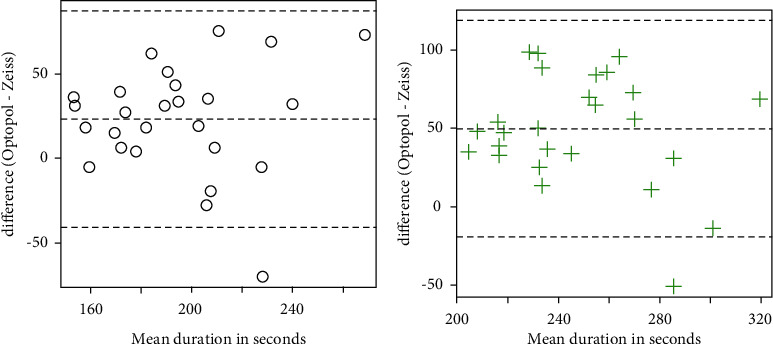
Bland–Altman plots for the time to complete the test (in seconds). For each subject, the mean of the measurement from the Zeiss and Optopol machines is taken and plotted on the *x*-axis. The *y*-axis is the difference between the Optopol and Zeiss measurements. The plot on (a) is for control subjects; the plot on (b) is for subjects with glaucoma.

**Figure 3:**
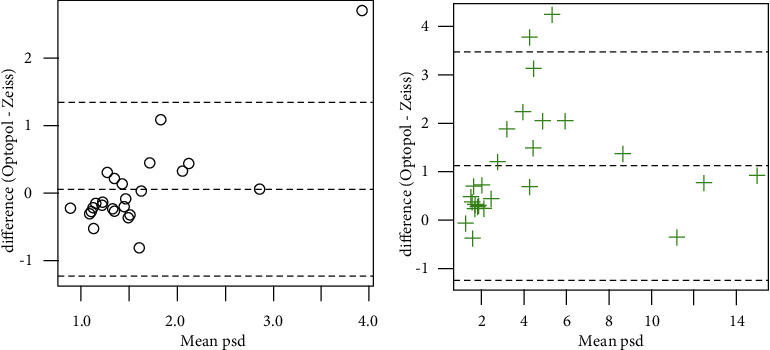
Bland–Altman plots for the visual field pattern standard deviations. For each subject, the mean of the measurement from the Zeiss and Optopol machines is taken and plotted on the *x*-axis. The *y*-axis is the difference between the Optopol and Zeiss measurements. The plot on (a) is for control subjects; the plot on (b) is for subjects with glaucoma.

**Figure 4:**
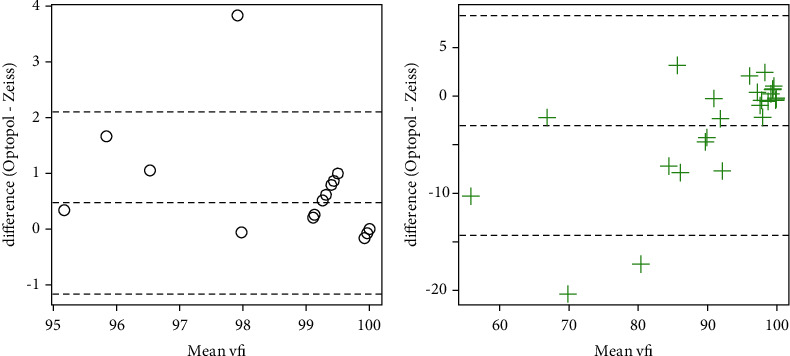
Bland–Altman plots for the visual field indices. For each subject, the mean of the measurement from the Zeiss and Optopol machines is taken and plotted on the *x*-axis. The *y*-axis is the difference between the Optopol and Zeiss measurements. The plot on (a) is for control subjects; the plot on (b) is for subjects with glaucoma.

The PSD and VFI data were neither normal nor amenable to transformation, so Wilcoxon signed-rank tests were used. There was insufficient evidence to detect a difference in PSD for control subjects (*v* = 200.5, *p*=0.53), but a significant difference was observed for subjects with glaucoma (*v* = 16, *p* < 0.001). Significant differences were detected for the VFI for both control (*v* = 10, *p*=0.002) and glaucoma subjects (*v* = 263.5, *p*=0.026). Information on whether the values for PSD and VFI given by the Optopol machine were higher or lower is listed in Table 2, but major points are summarized here: in the control subject population, 16 of 26 subjects (62%) had lower PSD values with Optopol when compared to Zeiss, whereas in the glaucoma subject population, 23 of 26 subjects (88%) had higher PSD values. Similarly, with VFI, 13 of 26 subjects (50%) had greater or equal VFI in the control subject population, whereas 18 of 26 subjects (69%) in the glaucoma population had lower VFI with Optopol. Bland–Altman plots are presented in Figures 1–4 depicting differences in mean MD, mean duration, mean PSD, and mean VFI between Optopol and Zeiss.

**Figure 5:**
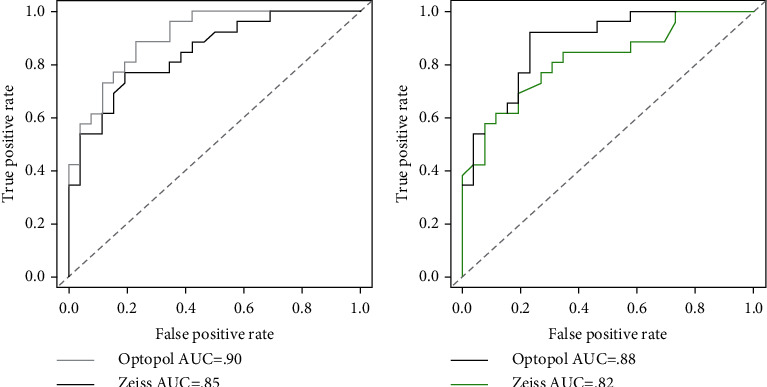
Receiver operating characteristic (ROC) curves for MD/PSD. ROC curves distinguishing between control and glaucomatous subjects based on MD (a) and PSD (b) indices from ZETA Fast (Optopol) vs. SITA Fast (Zeiss).

As a result of these patterns, the readings from the Optopol machine make it easier to distinguish between control and glaucomatous subjects than those from the Zeiss machine based on MD and PSD. These facts can be seen in receiver operating characteristic (ROC) curves (Figure 5). The areas under the ROC curves (AUC) were larger in MD (Optopol: AUC = 0.87; Zeiss: AUC = 0.84) and PSD (Optopol: AUC = 0.88; Zeiss: AUC = 0.82) (Figure 5).

Confusion matrices are presented in Table 3. Very few control subjects had MDs <−2. Thus, Optopol's accuracy (0.88) was not significantly greater than the no information rate (0.92, *p*=0.88) in this category, but neither was there evidence of bias (*p*=1.0). The sensitivity and specificity are 0.50 and 0.92, respectively. However, the data are more interesting in the glaucomatous subject population; Optopol's accuracy (0.92) was more informative than guessing the most prevalent category or no information rate (0.54, *p* < 0.001) for subjects with glaucoma. There is no significant evidence of bias (*p*=0.48). The sensitivity and specificity are 1.0 and 0.83, respectively.

## 4. Discussion and Conclusion

An automated perimeter produces a map of the differential light sensitivities at a range of eccentricities. This is compared with age-adjusted normal sensitivities (total deviation) and described in terms of the likelihood that each point falls within the normal range (total deviation probability plot). The field is then adjusted for overall depression to account for diffuse field loss that may be more likely due to refractive media opacity and thereby to highlight field loss from visual pathway pathology (pattern deviation) [16].

Both ZETA Fast (PTS) (Optopol Technology) and Humphrey SITA Fast (SFA) (Carl Zeiss Meditec) offer standardized perimeters of visual field-testing conditions. The equality in technical specification implies that the results obtained with one perimeter could be compared to those from another. Hence, one can assume that the differences demonstrated in this study originate from algorithm or strategy differences in ZETA Fast and SITA Fast tests, respectively.

The main goal of the ZETA Fast and SITA Fast strategies is to minimize test duration while maintaining clinical capabilities of threshold testing. When looking at differences in MD, the difference between the ZETA Fast and SITA Fast strategy is significantly within ±1 dB for the control subject population with a difference of 0.36 (*p*=0.006). Although the test results vary by greater than 1 dB between Optopol and Zeiss in glaucoma subjects (even after normalization) with a difference of log 1.11 dB (*p*=1.0, a model-predicted median difference of 3.0 on the original scale), there are however several data points on the Bland–Altman plot that reveal a tendency for Optopol to pick up a more negative MD value suggesting either a high false negative rate or, conversely, a higher sensitivity rate in detecting smaller, early visual field deficits when compared to Zeiss (Figure 1).

The testing time between Optopol and Zeiss favored Optopol with having a slightly quicker testing algorithm (Figure 2).

There is a similar pattern when looking at PSD indices which are similar between Optopol and Zeiss for control subjects but tend to skew positive in glaucoma subjects suggesting, again, either a higher sensitivity for true visual field deficits or a high false positive rate (Figure 3).

The above comparisons are clearer in Table 2 which is a depiction of the number of times the reading from the Optopol machine was less than, equal to, or greater than the readings from Zeiss for PSD and VFI indices. Generally, the PSD values were fairly balanced in control subjects, but typically higher in the Optopol measurements for glaucoma subjects. Likewise, for the VFI, the Optopol measurements were largely equal to or greater than Zeiss, amongst control subjects, but mostly lower in the glaucoma subject population (Figure 4).

The above statements regarding MD and PSD are better pointed out when looking at a receiver operating curve (ROC) analysis (Figure 5).

As mentioned, based on the scatterplot and Bland–Altman plots, Optopol tends to pick up a more negative MD value and higher PSD value in glaucomatous subjects. Thus, the MD and PSD values from Optopol better discriminate between control and glaucomatous subjects, as can be seen in the ROC analysis. In its entirety, this suggests that the ZETA Fast strategy is likely more sensitive in picking up earlier defects with a higher tendency for increased MD and PSD values with a lower VFI correlation. The fact that VFI is also higher in the control subject population again suggests that the strategy is superior and robust in identifying accidental invalid responses by subjects, leading to less nonspecific defects in healthy visual fields.

Another matter of interest is Optopol PTS ZETA Fast's ability to detect a MD of less than −2 while comparing its results to the Zeiss HFA SITA Fast strategy. This is an important aspect for a perimetric strategy, owing to its ability to pick up the earliest glaucomatous visual field defects with high sensitivity. As listed in the confusion matrices in Table 3, very few control subjects have MDs less than −2. As such, much information cannot be gleaned from this group and is clinically irrelevant. For subjects with glaucoma, Optopol was 100% sensitive and 83% specific in detecting subjects with early visual field deficits with MD less than −2 that were detected by Zeiss. Its accuracy was 92% (*p* < 0.001). Therefore, ZETA Fast is an equivalent perimetric strategy in accurately diagnosing early glaucomatous defects when compared to Zeiss HFA.

Based on our data, an apparent limitation of the ZETA Fast strategy was that the test took longer compared to SITA Fast. The ZETA Fast group was, on average, 23 seconds longer for control subjects (SD = 32.6, *p*=0.001) and 49 seconds for glaucoma subjects (SD = 35.5, *p* < 0.001), respectively.

Both ZETA Fast and SITA Fast are expeditious strategies which enable successful identification of glaucomatous defects. Our study has demonstrated that both algorithms offer the same high sensitivity of testing which has not been compromised by a longer Optopol test duration. Both strategies enabled effective identification of glaucomatous defects within 6 minutes, with the Zeiss machine obtaining results nearly 1 minute faster. Benefits of the ZETA Fast strategy include a possibility of obtaining higher peak PSD indices correlated with lower VFI in the glaucomatous subject making the perimeter an effective option for glaucoma screening and monitoring.

## Figures and Tables

**Table 1:** Characteristics of our sample, both overall and stratified by whether the subject had glaucoma.

	Overall	Stratified by glaucoma		*p*
		No	Yes	
*n*	52	26	26	
Age	56.98 (17.16)	46.42 (14.62)	67.54 (12.47)	<0.001
Gender				
F	30 (57.7)	17 (65.4)	13 (50.0)	0.4
M	22 (42.3)	9 (34.6)	13 (50.0)	
Race				
Unknown/others	4 (7.7)	1 (3.8)	3 (11.5)	0.58
Black	5 (9.6)	2 (7.7)	3 (11.5)	
White	43 (82.7)	23 (88.5)	20 (76.9)	
Ethnicity				
Hispanic	2 (3.8)	1 (3.8)	1 (3.8)	1.0
Non-Hispanic	49 (94.2)	25 (96.2)	24 (92.3)	
Unknown/others	1 (1.9)	0 (0.0)	1 (3.8)	

Categorical variables (e.g., gender) are represented by counts with percentages in parentheses. Age (a continuous variable) is represented by means with standard deviations in parentheses. Gender is tested with a chi-squared test; race and ethnicity are tested with Fisher's exact test. Age is tested with a *t*-test.

**Table 2:** The number of times (with the percentages in parentheses) that the reading from the Optopol machine was less than, equal to, or greater than the reading from the Zeiss machine.

	PSD		VFI	
	Normal	Glaucoma	Normal	Glaucoma
Optopol <	16 (62%)	3 (12%)	4 (15%)	18 (69%)
Optopol =	0 (0%)	0 (0%)	9 (35%)	0 (0%)
Optopol >	10 (38%)	23 (88%)	13 (50%)	8 (31%)

PSD readings are in the left two columns, with VFI readings in the right columns.

**Table 3:** Confusion matrices for Optopol's detection that MD <−2 as a prediction for Zeiss' detection of the same.

	Normal		Glaucoma	
	Zeiss MD <−2	Zeiss MD ≥−2	Zeiss MD <−2	Zeiss MD ≥−2
Optopol MD <−2	1	2	14	2
Optopol MD ≥−2	1	22	0	10

The matrix on the left pertains to control subjects, and that on the right pertains to subjects with glaucoma.

## Data Availability

The data are available through the NC Translational and Clinical Sciences (NC TraCS) Institute. Additionally, they can be obtained by our statistician Jeff Laux (lauxjp@live.unc.edu).
